# The Epidemiology of Needlestick and Sharp Injuries Among Healthcare Workers in a Secondary Care Hospital in Saudi Arabia: A Retrospective Study

**DOI:** 10.7759/cureus.58880

**Published:** 2024-04-23

**Authors:** Azer Rashidov, Husam Katib, Sarah K Alem, Faisal Al Harbi, Aminah Noor, Rosma Luna

**Affiliations:** 1 Infectious Disease, King Abdulaziz Hospital, Makkah, SAU; 2 Internal Medicine, Jacobs School of Medicine and Biomedical Sciences, Buffalo, USA; 3 Radiology, King Abdullah Medical City, Makkah, SAU

**Keywords:** needle stick and sharp injury, risk factors, healthcare work force, occupational risk exposure, infectious diseases

## Abstract

Introduction

Needlestick and sharp injuries (NSI) continue to pose a significant risk for healthcare workers (HCWs) at their workplace. The incidence rate of NSI in hospitals depends on multiple risk factors. This study aimed to analyze the epidemiological characteristics of NSI among HCWs and the risk factors influencing NSI rates and to provide further direction for NSI prevention in secondary care hospitals.

Methods

This study included all the NSI cases reported by HCWs in King Abdul Aziz Hospital, Makkah from 2005 to 2017. All the cases were recorded in the Exposure Prevention Information Network (EPINet™) database (International Healthcare Worker Safety Center, University of Virginia, Charlottesville, USA). The study was executed by using data loaded in the EPINet™^ ^Program, the hospital electronic recording system Medica Plus, and analyzed by the Statistical Package for the Social Sciences program (SPSS Inc. Released 2007. SPSS for Windows, Version 15.0. Chicago, SPSS Inc.).

Results

During the period of study, 524 NSI cases were reported. The mean incidence rate per 100 occupied beds with 95% CI was 25.43 (22.05-28.81) and a statistically insignificant decline in NSI incidence rate was observed from 2005 to 2017. The maximal annual incidence rate (35.63 per 100 occupied beds) was registered in 2010 and the minimal value (14.84 per 100 occupied beds) in 2013. Injuries were mainly reported in patient rooms/wards (30.2%) and most frequently by nurses (56.1%). The mean of incident reporting within 24 hours was 74.0, 95% CI (67.19-80.73). This rate showed a statistically significant (p=0.01) increasing trend of 5.0% per annum. The mean of identified source patients - 83.5, 95% CI (79.13- 87.23) - possessed an annual 2.1% rise during 2005-2017 which was statistically insignificant (p=0.7). Cases occurred after the use/before disposal of items in 45.0% of cases and during the use of items in 44.7%. Hollow-bore needles caused injuries in 46.5% of incidents. Blood sample taking - 23.2% and IV or arterial line insertion/removal/manipulation (19.1%) - presented exposure-prone procedures posing the highest risk.

Conclusions

The results of this study revealed a high rate of NSI in the hospital. NSI rate in hospitals was impacted by a group of related risk factors, particularly, the location of risk (patient room/ward, intensive care unit (ICU), and emergency room (ER) depending on job intensity, the kind and frequency of exposure-prone procedures (blood sample taking, IV or arterial line insertion/removal/manipulation) and handling of hollow-bore and solid needle connected to the main healthcare professional group at risk (nurses). Future direction in NSI prevention requires a complex approach of continuous staff education along with the usage of devices with safety features.

## Introduction

A needlestick and sharp injury (NSI) is the penetration of the skin by a needle or other sharp objects, which has been in contact with blood, tissue or other body fluids before the exposure. NSIs continue to pose a significant risk for healthcare workers (HCWs) at their workplace, causing serious aftermaths including long-term illnesses, disability and death for exposed HCWs and a substantial economic burden for the healthcare system as well. These injuries are primarily associated with occupational transmission of hepatitis B virus (HBV), hepatitis C virus (HCV), and human immunodeficiency virus (HIV). Still, they may be implicated in transmitting more than 20 other pathogens [1]. WHO stated in the World Health Report in 2002 that among the 35 million HCWs worldwide, about three million receive percutaneous exposures to bloodborne pathogens each year; two million of those to HBV, 0.9 million to HCV and 170,000 to HIV. These injuries may result in 15,000 HCV, 70,000 HBV and 500 HIV infections. More than 90% of these infections occur in developing countries. Worldwide, about 40% of HBV and HCV infections and 2.5% of HIV infections in HCWs are attributable to occupational sharps exposures [2]. In another study, it was estimated that annually there are 66,000 cases of HBV infection, 16,000 HCV infections and 1000 HIV infections worldwide among healthcare professionals, which result from NSIs and contact with blood and body fluids [3]. The Centers for Disease Control and Prevention (CDC) estimates that in the United States (US) each year 385,000 needlesticks and other sharps-related injuries are sustained by hospital-based healthcare personnel; an average of 1,000 sharps injuries per day [4]. The estimated annual incidence of needlestick injuries equals 100,000 in the United Kingdom, 700,000 in Germany, 29,719 in France, 28,200 in Italy, and 21,815 in Spain [5]. According to the reviewed national surveillance studies, NSI rates differed from country to country, particularly, in Saudi Arabia - 3.2 [6], Japan - 4.8 (for hospitals with 399 or fewer beds), 6.7 for hospitals with 400-799 beds and 7.6 for hospitals with 800 or more beds [7], France - 6.3 [8] and USA - 33.8 [9] injuries per 100 occupied beds. NSIs present an additional challenge: some cases remain underreported to employee health services. Globally two million HCWs are exposed to blood-borne pathogens annually and up to 75% (40-75%) of NSIs are not reported [2].

The incidence rate of NSIs and post-exposure prophylaxis outcomes in hospitals depend on multiple risk factors, including job category, the location where the exposure occurred, type of device, exposure-prone procedures, the time between the occurrence of NSI and its reporting, identification of the exposure source, etc. An effective NSI prevention strategy in the hospital requires a comprehensive approach to data collection, analysis and interpretation, evaluation of risk factors, rate monitoring, and designing of control measures. Despite the previous publications [6,10-12], there is a lack of research regarding the epidemiological features of NSI as well as risk factors impacting NSI rates in secondary care hospitals of the Western Region of the Kingdom of Saudi Arabia (KSA). This study aimed to analyze the epidemiological characteristics of NSI among HCWs as well as the risk factors influencing NSI rates and to provide further direction for NSI prevention in secondary care hospitals.

## Materials and methods

Methods

This retrospective study involved HCWs at 300 bedded secondary King Abdul Aziz Hospital located in Makkah, Saudi Arabia over 13 years from January 2005 to December 2017. This study included all the NSI cases reported by HCWs from 2005 to 2017. The study was executed by using data recorded in the Exposure Prevention Information Network (EPINet™) Program (International Healthcare Worker Safety Center, University of Virginia, Charlottesville, USA), the hospital electronic recording system Medica Plus as well as analyzed by the Statistical Package for the Social Sciences program (SPSS Inc. Released 2007. SPSS for Windows, Version 15.0. Chicago, SPSS Inc.).

Data collection

NSI surveillance in hospitals is based on a self-reporting system. Each report should be loaded into the EPINet™ database. EPINet™ is a software program that provides standardized methods for recording and tracking percutaneous injuries and blood and body fluid contacts, analyzing injury frequencies by attributes such as jobs, devices, and procedures as well as preparing monthly, quarterly and annual exposure reports. Each report includes employee and injury information sections. The employee information part comprises data about employee identification, age, gender and occupation. The injury information part consists of multiple data, particularly, the date and time of injury, the time of case reporting, patients, injury location, questions concerning how the injury occurred, if the injured worker was the original user, if the sharp item was contaminated, for what purpose the sharp object was originally used for, what type of device caused the injury, whether the sharp device causing injury has a safety device, injury depth and injury location. The hospital has an electronic health care recording system Medica Plus for HCWs and patients. All newly recruited employees are screened by the Employee Health Clinic to determine a serology status for HBV, HCV, and HIV. If susceptibility to HBV is defined, the HCW is advised to get the hepatitis B vaccine series that is fixed in the HCW's file. Data regarding the history of hepatitis B vaccination and the result of hepatitis B antibody level can be extracted from HCWs’ electronic file “Health Certificate” stored in the hospital’s electronic health care recording system, Medica Plus. HBV surface antigen (HBsAg), anti-HCV, and HIV test results of the source patients are available in the patient’s file and also stored in the hospital’s electronic health care recording system, Medica Plus. The source patient is considered unidentified if HCW was exposed to a patient who had no serology result at the time of injury or later due to fast discharge or death as well as if HCW was injured by a disposed device with unspecified source.

Data calculation, analysis and interpretation

The collected data of NSIs among the HCWs during the study period (from 2005 to 2017) were analyzed using the SPSS program (version 15.0). NSI incidence rate was calculated as the number of NSIs reported each year from 2005 to 2017 per 100 average daily hospital bed numbers. The values of epidemiological characteristics of NSIs among HWCs represented the mean for the 2005-2017 period of study. Trends of NSI incidence rate were determined using simple linear regression.

Ethical consideration

The highest level of objectivity in discussions and analyses throughout the research was maintained. Research was carried out based on informed consent. The privacy and anonymity of injured HCWs were of paramount importance. The ethics committee of King Abdul Aziz Hospital, Makkah issued clearance to enable access to the health workers’ records.

## Results

**Figure 1:**
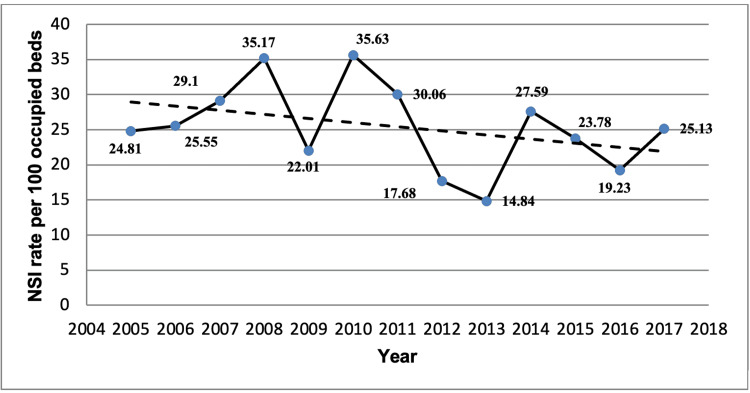
Incidence rate of NSI at King Abdul Aziz Hospital, Makkah during the period of 2005-2017. The solid line shows the annual incidence rate. The dotted line shows the linear simple regression. NSI: needlestick and sharp injury

During the period of the study, 524 cases of NSIs were reported. The mean incidence rate per 100 occupied beds with 95% CI was 25.43 (22.05-28.81). There was a negative drop or decline in the incidence rate from 2005 to 2017, although statistically insignificant (Figure 1).

The maximal annual rate was registered in 2010 - 35.63 per 100 occupied beds and the minimal value reported in 2013 - 14.84 per 100 occupied beds. A statistically significant increase in NSI rate was observed in 2010 (IRR, 0.62; CI 0.39-0.96, p=0.02) and 2014 (IRR 0.54; CI 0.32-0.90, p=0.01) in comparison with 2009 and 2013, respectively (Table 1).

**Table 1:** Distribution of NSIs by year (n=524) NSIs: needlestick and sharp injuries

Year	Frequency of recorded needle stick and sharp object injury	Average daily occupied hospital beds	NSI rate per 100 occupied hospital beds
2005	33	133	24.81
2006	35	137	25.55
2007	39	134	29.1
2008	51	145	35.17
2009	35	159	22.01
2010	57	160	35.63
2011	52	173	30.06
2012	32	181	17.68
2013	27	182	14.84
2014	40	145	27.59
2015	39	164	23.78
2016	35	182	19.23
2017	49	195	25.13

The most frequently reported exposures were observed among nurses (56.0%), medical doctors (9.0%), housekeepers (8.9%) and surgeons (7.8%). The rest of the exposures (17.2%) were reported by other exposed occupational groups. Most exposures occurred within the patient room/ward - 158 cases (30.2%), followed by the Intensive Care Unit - 17 (22.3%), Emergency Department - 101 (19.3%) and operating room - 53 cases (10.0%). The mean of incident reporting within 24 hours was 74.0, 95% CI (67.19-80.73). This rate showed a statistically significant (p=0.01) increasing trend of 5.0% per annum. The mean of identified source patients - 83.5, 95% CI (79.13-87.23) - possessed an annual 2.1% rise during 2005-2017 that was statistically insignificant (p=0.7). Concerning the mechanism of incidents, injuries mainly occurred after use before disposal of items in 45.0% of cases and during use of items in 44.7%. Hollow-bore needle (46.5%), solid needle (18.1%), surgical blade/scalpel (9.3%) and suture needle (9.3%) were those devices that mostly caused injuries while being handled by HCW, whereas the rest of the utilized sharps made up 18.3% (Table 2).

**Table 2:** Epidemiological characteristics of NSIs among HWCs of King Abdul Aziz Hospital, Makkah during 2005-2017 (n=524) NSIs: needlestick and sharp injuries; HCWs: healthcare workers

Variables	Frequency	(%)*
Job category of the injured worker		
Nurse	294	56
Medical doctor	47	9
Housekeeper	46	8.9
Surgeon	41	7.8
Technician (non-lab)	26	5
Nurse student	27	5.2
Dentist	23	4.4
Medical student	8	1.4
Laundry worker	4	0.9
Other attendants	8	1.5
Location of injury		
Patient room/ward	158	30.2
Intensive Care Unit	117	22.3
Emergency Department	101	19.3
Operating Room	53	10
Dental Outpatient Department	37	7.1
Hemodialysis Unit	34	6.6
Clinical Laboratory	8	1.5
Outpatient Department	6	1.1
Day Procedure Unit	3	0.6
Others	7	1.3
Incident reporting time		
Within 24 hours	388	74.0
Between 24-48 hours	61	11.7
After 48 hours	75	14.3
Identified source patient		
Yes	438	83.5
No	86	16.5
Type of device caused the injury		
Hollow-bore needle	244	46.5
Solid (hypodermic) needle	95	18.1
Suture needle	49	9.3
Surgical blade/scalpel	49	9.3
IV canula central line	21	4.1
Dental bur	8	1.5
Unknown device/not sure	4	0.8
Tooth extractor	3	0.6
Surgical scissor	3	0.5
Microtome	3	0.5
Probe tip	3	0.5
Metal brush	2	0.4
Bone cutter	2	0.3
Carrier elevator	2	0.3
Others	38	7.3
Time of injury occurrence		
After use, before disposal of the item	236	45
During the use of the item	234	44.7
After disposal of the item	28	5.4
Between steps of a multi-step procedure	17	3.2
While putting the item into the disposal container	9	1.7

The distribution of NSI cases according to the exposure-prone procedures and activities posing the highest risk was blood sample taking - 23.2%, IV or arterial line insertion/removal/manipulation - 19.1% and surgical procedure/wound care - 12.8% out of 524 reported cases (Table 3).

**Table 3:** Exposure-prone procedures causing NSIs NSIs: needlestick and sharp injuries; NG: nasogastric; ICT: intercostal catheter

Procedure	Frequency	%*
Blood sample taking	132	25.2
IV or arterial line insertion/removal/manipulation	112	21.4
Surgical procedure (e.g., all surgical procedures)	72	13.7
Subcutaneous or intramuscular injection	41	7.8
Dental procedure	31	5.9
Cleaning/transporting contaminated equipment	30	5.7
Blood sugar checking	29	5.5
Changing dressing/wound care	26	5.0
Manipulating blood tube/bottle/specimen container	22	4.2
Tube placement/removal/manipulation (e.g., chest endotracheal, NG, central line, ICT, urine catheter)	13	2.5
Dialysis procedure	4	0.8
Airway manipulation (e.g., suctioning airway, inducing sputum)	4	0.8
Other Irrigation procedure	8	1.6

## Discussion

Our study has detected that the mean incidence rate for the study period was 25.43, 95% CI (22.05-28.81). Two statistically significant maximal if to compare with previous years were observed in 2010 and 2014 with incidence rates of 35.63 and 27.59 per 100 occupied beds, respectively (Figure 1). The highest incidence rate was recorded in 2010 (35.63 per 100 daily occupied beds) while the lowest incidence rate was registered in 2013 (14.84 per 100 occupied beds) (Table 1). When comparing the NSI incidence rate in our hospital to other studies, it should be pointed out that our incidence rate was higher than those previously reported [6,7,10,13], almost identical to the 2005-2017 mean of EPINet that was 25.81, 95% CI (23.08-28.54) [14] as well as less than the corresponding rate reported from research involving HCWs at the University of Kentucky [15]. The lower NSI rate in comparison with the data of the University of Kentucky hospital mentioned in our findings can be explained by underestimation and underreporting of exposure by HCWs. Comparatively different NSI rate depends on variations in health care systems [11] as well as being impacted by organizational (hospital standards and policies), engineering (availability and access to instruments with safety features) and behavioural (recapping needles and deficiency in sharp disposal practice) risk factors [16] prevailed in different healthcare facilities.

Nurses were the most affected group among the HCWs (56.0%) followed by medical doctors (9.0%), housekeepers (8.9%) and surgeons (7.8%) (Table 2). This result ties well with previous studies wherein nurses got NSI more frequently than other professional groups [6,7,10,11]. It is important to note that the presented evidence relies on the fact that nursing staff represents the largest number of the workforce of any healthcare facility and their job description involves procedures with potential NSIs high risks, particularly blood sample taking, IV or arterial line insertion/removal/manipulation, subcutaneous or intramuscular injection, etc. Despite the widely accepted evidence of NSI's higher prevalence among nurses, some studies have reported physicians [16], surgeons [17] and housekeepers [13] as the more frequently injured groups. In our study physicians were the second most affected professional group. Housekeepers whose jobs do not require the routine use or handling of sharps were the third most impacted group indicating that the healthcare staff abuse the hospital’s Sharp Disposal policy. Improper sharp disposal by direct-care providers resulted in housekeeper exposure to the sharps while they collected waste. This finding is in accordance with studies that showed housekeepers as a group having a high exposure rate followed by doctors and nurses [13].

A study showed that the majority of reported cases, 429 (81.8%), happened in four locations: patient room/ward - 158 cases (30.2%), Intensive Care Unit - 117 (22.3%), Emergency Department - 101 (19.3%) and Operating Room - 53 cases (10.0%). The rest of the cases, 95 (18.2%) were reported in other 12 locations including the Dental Outpatient Department, Hemodialysis Unit, Clinical Laboratory, Outpatient Department, Day Procedure Unit, etc. (Table 2). Overall these findings are in line with the previous studies in Saudi Arabia [6,10] and the USA where during 2005-2017 the rate of NSI cases in patient rooms/wards ranged between 23.3 and 34.7 [14]. Our results with minor differences were similar to other studies where the main risk location for NSI incidents was in patient rooms, followed by the Operating Room, Emergency Department, and Outpatient clinics [9,13]. However, there are studies with different findings obtained that reported other areas with the highest incidence rates, particularly, the Emergency Department [18], the Operating Room [15] and the Dental Department [12]. Variations exist in the reporting of NSI from the same location of urban and community hospitals. In one study, HCWs reported injuries more frequently at the Emergency Department of urban academic medical centers when compared to the pooled community sites: 20.3 versus 5.9 per 100.000 patient visits (p<0.001) [16].

The trend of two rates indicating healthcare staff awareness, notably, incident reporting within 24 hours and identified source patients showed an annual increase of 5.0% and 2.1%, respectively. The mean of incident reporting within 24 hours was 74.0, 95% CI (67.19-80.73) and matched with data from another study that presented similar findings [13]. The mean of identified source patients equal 83.5, 95% CI (79.13-87.23) was higher than previously reported studies [10,19]. At the same time identified source patient mean during the same study period of EPINet was 93.9, 95% CI (93.10-94.68) [14]. These two rates as indicators of HWC awareness showed an increasing trend and could be attributed to the effect of educational programs conducted during the period of study. The comprehensive training program on “NSI prevention, proper sharp disposal and post-exposure prophylaxis” for newly recruited staff and refreshment courses for experienced HCWs have been conducted in the hospital since 2005. Moreover, since 2016 this course has been included in the BICSL (Basic Infection Control License) program and is mandatory for all HCWs once per two years.

Injuries occurred after use before disposal of items in 45.0% out of 524 reported cases (Table 2). Recapping of sharps is an important issue in healthcare facilities and due to this behavioral risk factor HCWs usually experience injuries after use before disposal of syringes. In the literature reviewed recapping data ranged from 9.7% [7] to 14.1% [13] which was indicated as an essential NSI risk factor during the use of items 44.7% of NSIs. These findings match with those results observed in earlier conducted studies [5,10,13], particularly, the percentage of NSI taking place during the use of items (44.7%) was compatible with the EPINet data reported for the last 13 years (2005-2017) ranging 38.6-52.1% [14].

Of the 524 documented cases of NSIs 46.5% were attributed to hollow-bore needles, 18.1% to hypodermic needles followed by surgical blade/scalpel and suture needle - 9.3% each, respectively (Table 2). Other handled sharps made up of 18.3%. The present findings seem to be consistent with other research which pointed out hollow-bore and hypodermic needles as the most frequent cause of NSI risk [13,20]. Injuries from hollow-bore needles, especially those used for blood collection, are a significant concern due to the probability of containing residual blood associated with an increased risk of HIV transmission [21]. Analysis of exposure-prone procedures defined blood sample taking (23.2%), IV or arterial line insertion/removal/manipulation (19.1%) and surgical procedure/wound care (12.8%) activities as the highest risk (Table 3).

The findings of this study suggest that the risk of exposure in locations with the highest incidence rate depends on job intensity, the kind and frequency of exposure-prone procedures performed by certain professional groups as well as, particular devices and sharp objects mostly used in such facilities. Exposure-prone procedure (blood sample taking and IV or arterial line insertion/removal/manipulation) ties perfectly with the previous findings, particularly, location (patient room/ward), job category (nurses) and type of device causing injury (hollow bore needle). Accordingly, as an aftermath, these groups of risk factors can determine the high incidence rate of NSI in hospitals. The result of this research is consistent with the idea of the previously published study [22].

The establishing comprehensive prevention program for reducing the risk of NSI considers all aspects of the work environment particularly those risk factors that prevailed in healthcare facilities. The key role of appropriate educational programs has been emphasized by several studies that reported a noticeable reduction in NSI rate [23]. Another study showed a significant increase in NSI rate after educational intervention owing to a wide range of knowledge and awareness about needle stick injuries [24]. Overall our findings showed a reduction in the incidence rate during 2005-2017 due to the conducted educational program and improved awareness of HCWs. However, at the same time, a periodic marked increase in NSI rate was observed in 2010 and 2014 in comparison with 2009 and 2013, respectively (Figure 1 and Table 1). The inculcation and wide use of devices with safety features in healthcare practice can be considered a promising aspect for effective prevention and steady reduction of NSIs. Implementation of educational programs along with the introduction of devices with safety features showed a tremendous fall in NSIs [25]. Therefore, the launching of this complex approach, particularly, the implementation of ongoing educational programs along with devices with safety features usage, should be promoted and suited to the healthcare facilities.

## Conclusions

The results of this study revealed a high rate of NSI in the hospital. NSI rate in hospitals was impacted by a group of related risk factors, particularly, the location of risk depending on job intensity, the kind and frequency of exposure-prone procedures (blood sample taking, IV or arterial line insertion/removal/manipulation) and handling of hollow-bore and solid needle connected to the main healthcare professional group at risk (nurses). Preventive measures protocol needs to be updated for a constant reduction of the NSI rate. Future direction in NSI prevention requires a complex approach of continuous staff education along with the usage of devices with safety features. This approach needs to be prioritized according to the defined group of risk factors determined in each facility.
